# Viral Evasion Strategies in Type I IFN Signaling – A Summary of Recent Developments

**DOI:** 10.3389/fimmu.2016.00498

**Published:** 2016-11-11

**Authors:** Katharina S. Schulz, Karen L. Mossman

**Affiliations:** ^1^Department of Pathology and Molecular Medicine, McMaster Immunology Research Centre, Institute for Infectious Disease Research, McMaster University, Hamilton, ON, Canada

**Keywords:** virus, type I interferon, evasion, innate immune signaling, NFκB

## Abstract

The immune system protects the organism against infections and the damage associated with them. The first line of defense against pathogens is the innate immune response. In the case of a viral infection, it induces the interferon (IFN) signaling cascade and eventually the expression of type I IFN, which then causes an antiviral state in the cells. However, many viruses have developed strategies to counteract this mechanism and prevent the production of IFN. In order to modulate or inhibit the IFN signaling cascade in their favor, viruses have found ways to interfere at every single step of the cascade, for example, by inducing protein degradation or cleavage, or by mediate protein polyubiquitination. In this article, we will review examples of viruses that modulate the IFN response and describe the mechanisms they use.

## Introduction

The mammalian immune system evolved to detect and fight viral infections effectively. The induction of type I interferon (IFN), predominantly IFN-α and IFN-β, forms the first line of defense. The type I IFN response consists of two parts. First, the cell produces type I IFN, when triggered by a viral stimulus. The IFN is then secreted and, in the second part of the response, it is sensed by the producing, as well as neighboring cells, resulting in the production of IFN-stimulated genes (ISGs) [reviewed in Ref. (1)].

Viruses, which have coevolved with their host, develop strategies to counteract the signaling cascades of the innate immune system and ensure their replication. Recently, several reviews were published, describing the innate immune evasion strategies of individual viruses or virus families, such as influenza virus (2, 3), Phleboviruses (4), Herpes viruses (5–7), Coronaviruses severe acute respiratory syndrome (SARS) and middle east respiratory syndrome (MERS) (8), human immunodeficiency virus (HIV) (9, 10), as well as multiple RNA viruses (11, 12). Moreover, there are recent articles that review how viruses prevent detection by pathogen recognition receptors (PRRs) (13, 14) and how viruses modulate innate immune signaling by use of viral deubiquitinases (15).

In this review, we will compare the different strategies viruses have developed to suppress innate immune signaling of individual components of the innate immune signaling cascade. Due to the tremendous amount of data in this field, we will focus on recent discoveries. Older studies were summarized in Ref. (16, 17).

## Virus Recognition

Invading viruses are recognized by PRRs [reviewed in Ref. (14)]. The most important viral markers for the innate immune system are viral nucleic acids. The detection of viral DNA through the cGAS-Sting pathway and the counter measurements taken by viruses have been reviewed recently (18) and are not part of this review.

Viral RNAs, which are mostly double-stranded (ds-)RNA, are recognized by three PRRs: the endosomal toll-like receptor 3 (TLR3), the cytoplasmic retinoic acid-inducible gene I (RIG-I)-like receptors (RLRs), and the nucleotide-oligomerization domain (NOD)-like receptors (NLRs) (19). TLR3 and the RLRs are important for inducing the type I IFN response, whereas NLRs have been shown to regulate interleukin-1β (IL-1β) maturation through activation of caspase-1 (20). The group of RLRs consists of RIG-I, melanoma differentiation-associated gene 5 (MDA5), and laboratory of genetics and physiology 2 (LGP2). The three receptors have a similar structure, all containing a caboxy-terminal domain, which functions as a repressor domain (RD) in RIG-I and LGP2 (21) and a central helicase domain, but LGP2 lacks the caspase activation and recruitment domains (CARDs) that function in signaling [reviewed in Ref. (19, 22)]. Both the helicase and the carboxy-terminal domain are required for RNA binding. RIG-1 and MDA-5 detect specific viral RNA PAMPs, while LGP2 negatively regulates RIG-I signaling and promotes RNA binding to MDA5 [reviewed in detail in Ref. (14)].

In unstimulated cells, RIG-I and MDA-5 are kept in a repressed state due to phosphorylations on serine and threonine residues in the CARDs and carboxy-terminal domains (23, 24). Upon binding of RNA, both RIG-I and MDA-5 undergo conformational changes, resulting in release of their CARDs (25, 26). Recruited phosphatases remove the phosphate residues, and E3 ubiquitin ligases attach Lys63-linked ubiquitin polymers onto the CARDs and C-terminal domain of RIG-I, which are important for RIG-I tetramerization (27–31).

RNA-bound RIG-1 then interacts with 14-3-3ε, a mitochondrial trafficking protein, and the TRIM25 ubiquitin ligase, which together transport RIG-I to the mitochondria (32). There the CARDs of RIG-I or MDA-5 interact with the CARD of the mitochondrial activator of virus signaling (MAVS, also known as IPS-1, VISA, and Cardif), which is an essential signaling adaptor protein. The activation of MAVS has recently been reviewed in detail in Ref. (33).

**Figure 1:**
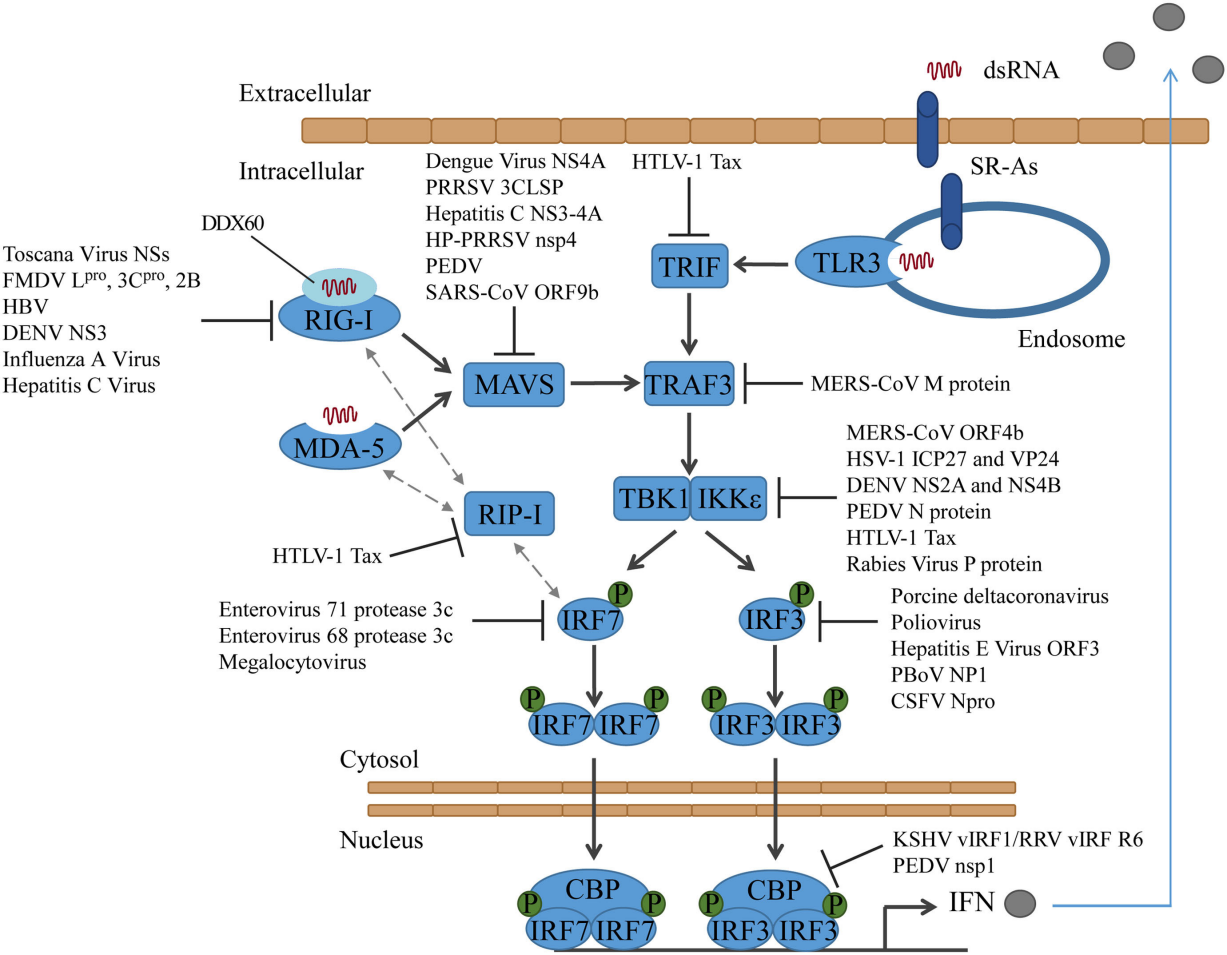
**Activation of interferon regulatory factors and the counteractions taken by viruses**. DsRNA is sensed by PRRs, which results in the activation of different adaptor proteins and the recruitment of TRAF3. TBK1 and/or IKKε are activated and phosphorylate IRF3 and/or IRF7, which then translocate into the nucleus to induce type I IFN expression. CSFV, classical swine fever virus; DENV, dengue virus; FMDV, foot-and-mouth disease virus; HBV, hepatitis B virus; HP-PRRSV, highly pathogenic porcine reproductive and respiratory syndrome virus; HSV-1, herpes simplex virus type 1; HTLV-1, human T-cell lymphotropic virus type I; KSHV, Kaposi’s sarcoma-associated herpesvirus; MERS-CoV, middle east respiratory syndrome coronavirus; PBoV, porcine bocavirus; PEDV, porcine epidemic diarrhea virus; PRRSV, porcine reproductive and respiratory syndrome virus; RRV, rhesus macaque rhadinovirus.

TLR3 interacts with TRIF, which serves as a molecular platform and forms physical interactions with several adaptor molecules (34). By interacting with upstream adaptors, TRIF undergoes conformational changes and recruits the downstream TNF receptor-associated factor (TRAF)3 and TRAF6 [reviewed in Ref. (35)]. The kinase receptor-interacting protein-1 (RIP-1) is part of both the signaling pathways downstream of TLR3 and RIG-I. It can interact with TRIF to induce NFκB activation (36). Moreover, the dsRNA-activated TLR3 can recruit TRIF, RIP-1, and Caspase-8 and induce apoptosis (37). Also, RIP-1 and its adaptor protein Fas-associated protein with death domain (FADD) are part of the signaling cascade downstream of RIG-I and MDA-5 and involved in the activation of the transcription factors interferon regulatory factor (IRF)3 and IRF7 (38). TRAF3 serves as a linker between the upstream adaptor proteins (TRIF or MyD88 for TLRs and MAVS for RLRs) and the downstream signaling kinases TBK1/IKKε or IRAK1/IKKα. The recruitment of TRAF3 to the TLR or RLR signaling complexes activates the E3 ligase activity of TRAF3, which then catalyzes its own K63-linked ubiquitinylation. Subsequent TRAF3 activates TBK1/IKKε or IRAK/IKKα [reviewed in Ref. (39)] (Figure 1).

Viruses target RIG-I directly or indirectly to block the type I IFN response. The phlebovirus Toscana Virus expresses a non-structural protein, which directly interacts with RIG-I and induces its proteasomal degradation (40, 41). Foot-and-mouth disease virus (FMDV) proteins L^pro^, 3C^pro^, and 2B increase the RIG-I mRNA expression but decrease the protein expression of RIG-I. L^pro^ and 3C^pro^ both induce RIG-I degradation, whereas the mechanism of how 2B reduces RIG-I protein levels has not been solved yet (42). Other viruses target RIG-I indirectly. Hepatitis B virus (HBV) induces miR146a, which then posttranscriptionally inhibits the expression of RIG-I and suppresses the production of type I IFN (43).

The dengue virus NS3 protein binds to 14-3-3ε and prevents the translocation of RIG-I to MAVS. The binding site on NS3 is a highly conserved phosphomimetic motif, which was verified by generation of a virus containing a mutation in this motif (44).

It has been proposed that in certain cell types RIG-I requires sentinels, such as the protein DDX60, which associates with RIG-I and promotes the RIG-I RNA-binding activity (45, 46). Other studies question DDX60 acting as a broadly active enhancer of antiviral responses (47, 48) and instead suggest that DDX60 only functions in the antiviral response to specific viruses, such as hepatitis C virus (47). However, there are data indicating that influenza A virus and hepatitis C virus attenuate IFNβ-promoter activation by targeting the sentinel DDX60. Both viruses activate the epidermal growth factor (EGF) receptor, which in turn phosphorylates DDX60 on Tyr-793 and Tyr-796. This results in the attenuation of DDX60-dependent RIG-I activation. In addition, independent of its role as sentinel for RIG-I viral RNA recognition, DDX60 plays a role in viral RNA degradation (46) (Figure 1).

Mitochondrial activator of virus signaling is blocked by different viruses in various ways. The dengue virus protein NS4A targets MAVS, and the interaction prevents the binding of MAVS to RIG-I (49). The porcine reproductive and respiratory syndrome virus (PRRSV) 3C-like protease (3CLSP), by contrast, cleaves MAVS in a proteasome- and caspase-independent manner at Glu268 (E268/G269). Both cleavage products fail to activate the type I IFN response (50). Likewise, the hepatitis C virus protein NS3-4A (51, 52), as well as the highly pathogenic porcine reproductive and respiratory syndrome virus (HP-PRRSV) protein nsp4 (53) have been shown to cleave MAVS and block RLR signaling. The porcine epidemic diarrhea virus (PEDV) also targets MAVS in small intestinal epithelial cells (IECs). However, the exact mechanism has not been solved yet (54) (Figure 1).

The SARS coronavirus protein ORF9b not only influences antiviral signaling but also alters host cell mitochondria morphology by inducing degradation of the dynamin-like protein (DRP1). MAVS becomes concentrated into small puncta in the presence of ORF9b (55). In addition, ORF9b triggers K48-linked ubiquitinylation of MAVS, by targeting the poly(C)-binding protein 2 (PCBP2) and the HECT domain E3 ligase AIP4. Under normal conditions, PCBP2 controls MAVS levels by linking the AID4 E3 ubiquitin ligase with MAVS (56). In addition to MAVS, also the levels of TRAF3 and TRAF6 are reduced by ORF9b. However, it is unlikely that TRAF3 and TRAF6 are targeted directly. More likely, they are degraded due to their interaction with MAVS (55) (Figure 1).

Human T-cell lymphotropic virus type I (HTLV-1) protein Tax disrupts innate immune signaling in multiple ways: it binds to the RIP homotypic interaction motif (RHIM) domains of RIP-1 and disrupts the interaction between RIP-1 and RIG-I or MDA-5 and the activation of the type I IFN promoter. Tax also binds to TRIF and thereby interrupts the TLR3 signaling cascade. Finally, Tax blocks the association between RIP-1 and IRF7, which resulted in repression of the IRF7 activity (57) (Figure 1).

Middle East respiratory syndrome coronavirus M protein interacts with TRAF3 and disrupts the interaction between TRAF3 and TBK1, which ultimately leads to a reduced IRF3 activation. For the interaction with TRAF3, the N-terminal transmembrane domain of the MERS-CoV M protein is sufficient (58), similar to what has been shown for SARS-CoV before (59) (Figure 1).

## Activation of Transcription Factors and IFN Transcription

**Figure 2:**
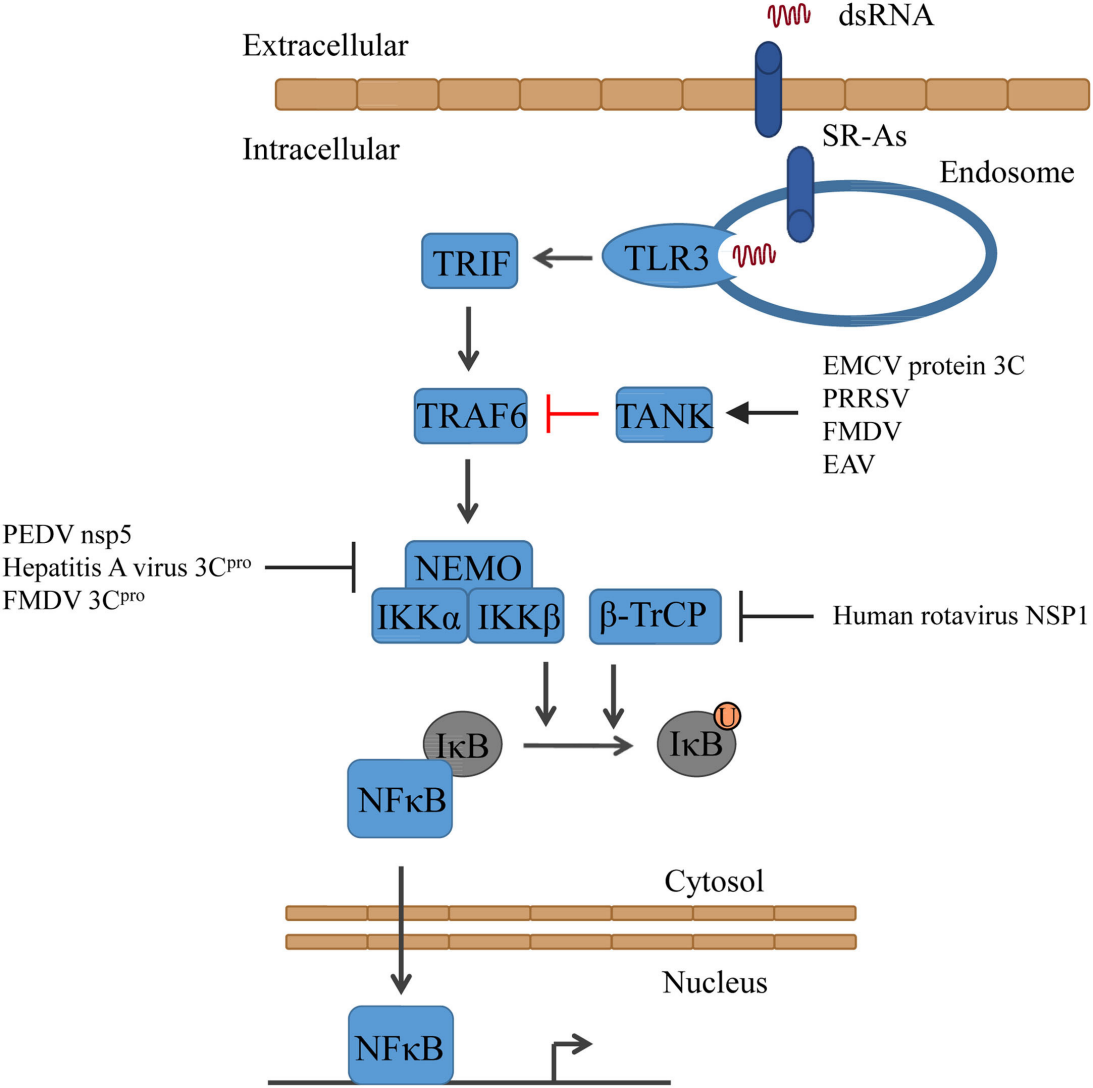
**Activation of NFκB signaling and the counteractions taken by viruses**. Triggering of TLR3 results in the activation of first TRAF6 and subsequently of IKK (consisting of NEMO, IKKα, and IKKβ). Together with β-TrCP, IKK mediates the ubiquitinylation of IκB, resulting in the release of NFκB. EAV, equine arteritis virus; EMCV, encephalomyocarditis virus; FMDV, foot-and-mouth diseases virus; PEDV, porcine epidemic diarrhea virus; PRRSV, porcine reproductive and respiratory syndrome virus.

Triggering of the TLR3- and RLR-signaling cascade results in the activation of the transcription factors NFκB and IRF3/IRF7. In its inactive state, the transcription factor NFκB is complexed with its inhibitor IκB (60). Upon stimulation, IκB is phosphorylated by the IκB kinase (IKK) complex, which is composed of two catalytic subunits, such as IKKα and IKKβ, and a regulatory subunit, such as NFκB essential modulator (NEMO) (61). The phosphorylation of IκBα induces its polyubiquitination through the E3 ubiquitin ligase β-transducin repeat-containing protein (β-TrCP) and subsequent proteasomal degradation (62), allowing NFκB to translocate into the nucleus and induce the expression of target genes (63) (Figure 2).

Encephalomyocarditis virus (EMCV) protein 3C cleaves TRAF family member-associated NFκB activator (TANK), which inhibits TRAF6-mediated NFκB activation, on Gln291. As a result, NFκB is activated and the unstable C-terminal fragment of TANK is subjected to proteasomal degradation (64). Also, other viruses express proteases that cleave TANK, although on other residues, such as porcine reproductive and respiratory syndrome virus (PRRSV) (TANK is cleaved by Nsp4), FMDV (protease 3C cleaves TANK), and equine arteritis virus (EAV) (TANK is cleaved by Nsp4). Thus, TANK seems to be a common target of several positive RNA viral proteases (64) (Figure 2).

Several viruses have been shown to disrupt IFN signaling by cleaving NEMO. PEDV 3C-like protease, nsp5, cleaves NEMO at Gln231 (65), whereas the hepatitis A virus 3C protease (3C^pro^) cleaves NEMO at Gln304 (66) and the picornavirus FMDV protease 3C^pro^ at Gln383, removing the C-terminal zinc finger domain from the protein (67). The human rotavirus has developed another way. Its non-structural protein 1 (NSP1) has been shown to inhibit the NFκB pathway by degrading β-TrCP and consequently stabilizing IκB (68) (Figure 2).

TANK-binding kinase 1 (TBK1) and inhibitor of κB kinase ε (IKKε) are classified as non-canonical serine/threonine kinases and are both able to induce IRF3 and IRF7 phosphorylation and subsequent dimerization (69–72). However, while TBK1 is constitutively expressed in most cell types, the expression of IKKε is more restricted (73). Upon stimulation, TBK1 and IKKε are recruited by adaptor proteins to signaling complexes to be activated by phosphorylation on Ser172 and both have been shown to be subjected to K63-linked polyubiquitination [reviewed in Ref. (73, 74)]. For TBK1, K63-linked polyubiquitination seems to be important for TLR- and RLR-induced IFN production, as ubiquitin chains might serve as a platform for the assembly of TBK1 signaling complexes. Moreover, deubiquitinases are able to terminate the TBK1-mediated pathway by cleaving the K63-linked ubiquitin chains [reviewed in Ref. (74, 75)]. Activated TBK1/IKKε phosphorylates IRF3 and/or IRF7 in the cytosol at specific serine residues. This phosphorylation results in homo- or heterodimerization of IRF3 and IRF7 and nuclear translocation (76, 77). Interestingly, while IRF3 is constitutively expressed, IRF7 is expressed at low levels in most cell types and expression is induced upon IFN signaling. Therefore, in most cells, IRF7 strongly enhances the production of IFN [reviewed in Ref. (78)]. Once phosphorylated IRF3 and/or IRF7 dimers have translocated into the nucleus, they bind to the transcription coactivator CREB-binding protein (CPB)/p300 (79, 80). Together with other factors, such as NFκB, they form the enhanceosome on the IFNβ promoter and induce the expression of type I IFN [reviewed in Ref. (76)].

The viral proteins that target TBK1 act by either blocking activation of TBK1 by MAVS or by inhibiting activation of IRF3 by TBK1. The MERS-CoV protein ORF4b blocks IFNβ production by binding to TBK1 and IKKε and suppressing the formation of a MAVS/IKKε complex (81). In addition to inhibiting TBK1/IKKε activation, ORF4b can also inhibit the production of IFNβ in the nucleus; however, the mechanism has not been solved yet (81). Recently, two herpes simplex virus proteins have been shown to target TBK1/IKKε and inhibit the phosphorylation of IRF3: ICP27 (82) and VP24 (83). Also, dengue virus serotype 4 non-structural proteins NS2A and NS4B, as well as the NS2A and NS4B proteins of other Dengue viruses, inhibit the phosphorylation of TBK1 (84) and PEDV N protein has been shown to interact with TBK1, hampering the association of TBK1 with IRF3 and preventing the activation of IRF3 activation (85). The human T-cell leukemia virus type 1 oncoprotein Tax has been shown to also interact with TBK1. However, studies came to contradicting results on how that influences the production of IFNβ. While one group showed that Tax activates TBK1 and the production of IFNβ (86), another group showed that Tax suppresses the IFN production by interaction with TBK1 (87). Interestingly, when a recent study tested how the rabies virus P protein of street strains behaves compared to laboratory-adapted strains with regard to the induction of type I IFN, they found that both street strains and laboratory strains inhibit TBK1-mediated signaling, but only the P protein of street strains also interacts with and inhibits IKKε-inducible IRF3-dependent IFNβ expression (88) (Figure 1).

Interferon regulatory factor 3 is targeted by many viruses to impair innate immune signaling. Most viruses inhibit the phosphorylation and thereby also the dimerization and translocation of IRF3, such as the porcine deltacoronavirus (89) or poliovirus (90). Hepatitis E virus protein ORF3 also suppresses IRF3 phosphorylation, but in an indirect way. It activates the signal regulator protein α (SIRP-α), which negatively regulates type I IFN induction (91). In contrast, porcine bocavirus (PBoV) NP1 protein does not affect IRF3 expression, phosphorylation, or nuclear translocation. Instead, it interacts with the DNA-binding domain of IRF3 and inhibits the DNA-binding activity (92). A very interesting way of how to circumvent the host innate immune response was found when studying gammaherpesviruses Kaposi’s sarcoma-associated herpesvirus (KSHV) and rhesus macaque rhadinovirus (RRV). They express several viral homologs to the IRFs, called viral IRFs (vIRFs). These vIRFs have found multiple ways to suppress type I IFN production. For KSHV, different strategies have been reviewed in Ref. (6). Recently, the RRV vIRF R6 has been shown to interact with the transcriptional coactivator CBP in the nucleus, similar to the KSHV vIRF1. As a result, CBP cannot form a complex with the phosphorylated IRF3, and the IFN expression is not induced (93–95). Interestingly, RRV R6 is the first vIRF for which an association with the viron could be shown. Therefore, vIRF V6 can shut down the type I IFN response shortly after the cell was infected, rendering the cell more susceptible to infection (95). The PEDV protein nsp1 also targets CBP. Nsp1 induces CBP degradation in a proteasome-dependent manner and thereby interrupts enhanceosome assembly and the production of type I IFN (96) (Figure 1).

For most of these interactions, the molecular mechanisms have not been unraveled yet. A protein that has been shown to interact with and induce proteasomal degradation of IRF3 some time ago is classical swine fever virus (CSFV) Npro (97, 98). Recently, the molecular mechanism has been published. IRF3 and Npro interact direct and form a soluble 1:1 complex. Moreover, it was shown that Npro interacts with the full-length IRF3, not with individual domains, and that Npro binds the constitutively active form of IRF3 in the presence of CPB. Thus, Npro interacts with both the monomer and the active IRF3 dimer and likely targets both species for ubiquitinylation and proteasomal degradation (99).

Interferon regulatory factor 7 is targeted by two human enteroviruses, such as enterovirus 71 and enterovirus 68. They downregulate IRF7 by cleaving it with their protease 3c, leaving the cleavage products unable to induce IFN expression. While enterovirus 71 cleaves IRF7 once at Gln189–Ser190 (100), Enterovirus 68 cleaves it twice, the cleavage sites being Gln167 and Gln189 (101). Moreover, megalocytivirus, a DNA virus that infects marine and freshwater fish, induces the expression of the host microRNA pol-miR-731, which then specifically suppresses the expression of IRF7 (102) (Figure 1).

## Type I IFN Signaling

The type I IFNs act in an autocrine, paracrine, or systemic manner to stimulate antiviral responses. They are recognized by the IFNα/β receptor (IFNAR), which consists of the subunits IFNAR1 and IFNAR2 expressed on virtually all cell types (103). The interaction of type I IFN with the receptor results in the phosphorylation and activation of the IFNAR1- and IFNAR2-associated tyrosine kinases tyrosine kinase 2 (TYK2) and Janus kinase 1 (JAK1), which then phosphorylate IFNAR tyrosine residues, resulting in the recruitment and activation of signaling molecules, such as the signal transducer and activator of transcription (STAT) family of transcription factors (104, 105). Upon activation, STAT1 and STAT2, together with IRF9, form the IFN-stimulated gene factor 3 (ISGF3), which then translocates into the nucleus to induce transcription of ISGs [reviewed in detail in Ref. (106–108)].

**Figure 3:**
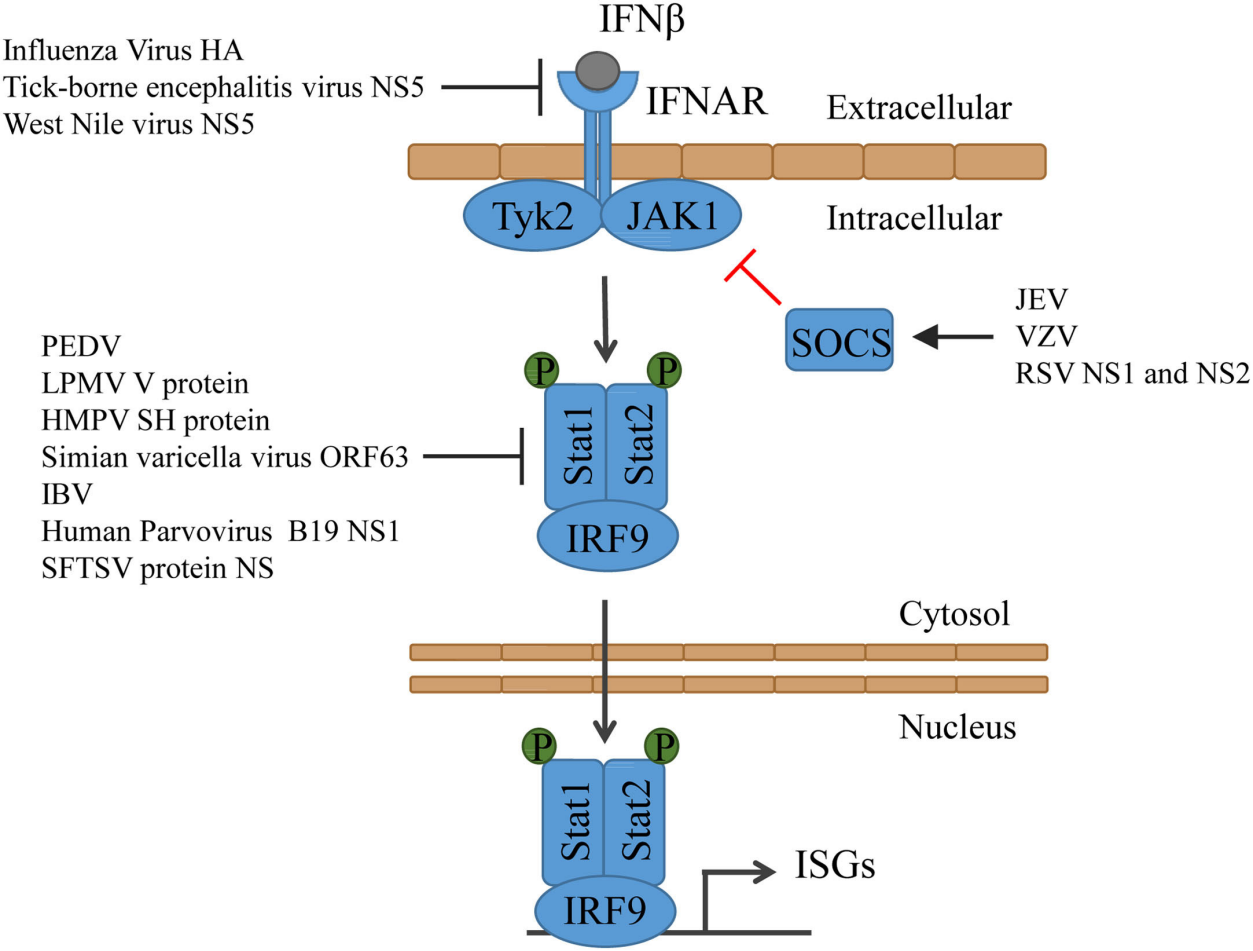
**Type I IFN signaling and the counteractions taken by viruses**. IFN binds to its receptor and thereby activates Tyk2 and Jak1, which then phosphorylate Stat1 and Stat2. Together with IRF9, Stat1 and Stat2 form the ISGF3, which translocates into the nucleus and induces the expression of ISGs. HMPV, human metapneumovirus; IBV, infectious bronchitis virus; JEV, Japanese encephalitic virus; LPMV, La Piedad Michoacán Mexico Virus; PEDV, porcine epidemic diarrhea virus; RSV, respiratory syncytial virus; SFTSV, severe fever with thrombocytopenia syndrome virus; VZV, varicella-zoster virus.

Several viruses target IFNAR to prohibit IFN binding and signaling. Influenza virus induces the degradation of IFNAR1. Hemagglutinin (HA) triggers the phosphorylation and ubiquitinylation of IFNAR1, thus promoting protein degradation (109). Encephalitic Flaviviruses, such as tick-borne encephalitis virus or West Nile virus, inhibit IFNAR1 surface expression. Their protein NS5 binds the cellular dipeptidase prolidase (PEPD), which is involved in IFNAR1 maturation and accumulation, activation of IFNβ-stimulated gene induction, and IFN-dependent viral control. This interaction inhibits IFNAR1 intracellular trafficking and glycosylation but does not promote IFNAR1 degradation (110) (Figure 3).

Both STAT1 and STAT2 are targeted by many viruses to suppress ISG induction. PEDV induces Stat1 ubiquitinylation and targets it for degradation in the proteasomes (111). Some viruses evolved to prevent the phosphorylation of Stat1 or Stat2. The paramyxovirus La Piedad Michoacán Mexico Virus (LPMV) V protein binds to Stat2 and prevents the type I IFN-dependent phosphorylation and nuclear translocation of Stat1 and Stat2 (112). Similarly, human metapneumovirus (HMPV) protein SH impairs Stat1 expression, phosphorylation, and activation (113). Simian varicella virus not only inhibits Stat2 phosphorylation but also promotes degradation of IRF9 in a proteasome-dependent manner through its protein ORF63 (114). Also, infectious bronchitis virus (IBV) inhibits phosphorylation and nuclear translocation of Stat1. However, despite detailed analyses, it is unclear which viral protein is responsible. It was, however, shown that the accessory protein 3a contributes to IBV resistance to type I IFN, although the target is unknown as well (115). In case of the human Parvovirus B19, it becomes evidently clear that both the virus and the immune system constantly evolve to prevail. While its protein NS1 suppresses Stat phosphorylation, the immune system senses the protein and triggers the production of type I IFN (116). SFTSV, an emerging tick-borne pathogen, developed multiple ways to prevent ISG induction. The viral non-structural protein NS impairs Stat1 expression, phosphorylation, and activation (117) and interacts with STAT2 and sequesters STAT1 and STAT2 into viral inclusion bodies, where they are trapped (118) (Figure 3).

The JAK-STAT signal transduction pathway is negatively regulated by the suppressor of cytokine signaling (SOCS) family of proteins in form of a classical feedback loop (119, 120). Some viruses induce the expression of SOCS to take advantage of this mechanism to minimize the induction of ISGs. Japanese encephalitic virus (JEV) downregulates the expression of microRNA miR-432, which then results in upregulated SOCS5 levels (121). Varicella-zoster virus (VZV) infection induces the expression of SOCS3 (122) and respiratory syncytial virus (RSV) non-structural proteins NS1 and NS2 induce upregulation of SOCS1 and SOCS3, which also inhibited the induction of chemokines (123) (Figure 3).

## Host Shut Off

Viruses fully depend on the translation machinery of the host cell for replication. Accordingly, they have evolved multiple ways to hamper host protein synthesis [reviewed in Ref. (124)]. One way is to shut off host protein synthesis. For some time, it was thought that Gamma- and Deltacoronaviruses do not induce host shutoff, such as Alpha- and Betacoronaviruses do. However, a recent study showed that the infectious bronchitis Gammacoronavirus induces host shutoff using its protein 5b. It seems like 5b is a functional equivalent of nsp1, the host shutoff protein of Alpha- and Betacoronaviruses (125).

## Conclusion

Viruses evolved to have various strategies to circumvent the innate immune response by blocking the production of type I IFN or the expression of ISGs. While these diverse strategies may appear contradictory between viruses, several factors require consideration. For example, the use of clinical isolates versus laboratory-passaged strains could yield different results, particularly with RNA viruses that rapidly accumulate mutations due to error-prone RNA-dependent RNA polymerases. Moreover, the choice of cell line can greatly influence experimental outcomes, as many immortalized or transformed continual cell lines harbor mutations in critical innate immune signaling (126). Likewise, the use of genetic knockout versus knockdown cell lines or organisms can influence experimental outcomes, as can the experimental procedures themselves, particularly when endogenous interactions are disrupted with the use of overexpression approaches.

Studying the mechanisms used by viruses to prevent an immune response is of great importance for the development of new strategies to limit the sequelae of viral infections. Identification of key immune evasion proteins allows development of antivirals to target these proteins. Alternatively, identification of key cellular antiviral pathways allows development of strategies to enhance these pathways to overwhelm incoming viruses. Information on key immune evasion factors further facilitates the engineering of safe and effective vaccine strains and designing strategies to target new emerging viruses from the same or closely related family.

## Author Contributions

KS and KM conceptualized the scope of the review article. KS wrote the review with input from KM.

## Conflict of Interest Statement

The authors declare that the research was conducted in the absence of any commercial or financial relationships that could be construed as a potential conflict of interest.
